# Development of a 5-HT_7_ receptor antibody for the rat: the good, the bad, and the ugly

**DOI:** 10.1007/s00210-023-02482-w

**Published:** 2023-04-18

**Authors:** Janice M. Thompson, Will Tragge, Emma D. Flood, Stefan Schulz, Erika Lisabeth, Stephanie W. Watts

**Affiliations:** 1 Department of Pharmacology and Toxicology, Michigan State University, 1355 Bogue Street, Rm B445, East Lansing, MI 48824-1317 USA; 2 Department of Pharmacology and Toxicology, Jena University Hospital, 07747 Jena, Germany; 3 7TM Antibodies, 07745 Jena, Germany

**Keywords:** GPCR antibody, 5-HT, 5-HT_7_ receptor, Westerns, Immunohistochemistry

## Abstract

Our laboratory has a vested interest in measuring the location and expression of the 5-hydroxytryptamine (5-HT, serotonin) 7 (5-HT_7_) receptor in the rat. Determining tissue-specific receptor expression would aid in validating understood and potentially new tissues that support the 5-HT_7_ receptor-mediated fall in blood pressure, an event we are committed to understand. We contracted with 7TM Antibodies to develop deliberately and rigorously a rat 5-HT_7_ (r5-HT_7_) receptor specific antibody. Three antigens, two targeting the third internal loop and one the C terminus, were used in three rabbits to generate antibodies. As a positive control, HEK293(T or AD) cells were transfected with a plasmid for the r5-HT_7_ receptor also expressing a C terminus 3xFLAG tag. Naïve rat tissues were also used in Western and immunohistochemical analyses. Nine antibodies (3 from three different rabbits) detected a ~ 75 kDa protein absent in homogenates of vector control HEK293T cells. Only antibodies that recognized the C terminus of the 5-HT_7_ receptor [ERPERSEFVLQNSDH(Abu)GKKGHDT; antibodies 3, 6, and 9] positively and concentration-dependently identified the r5-HT_7_ receptor expressed in Westerns of transfected HEK293T cells. These same C terminus antibodies also successfully detected the r5-HT_7_ receptor in immunocytochemical test of the transfected HEK293AD cells, colocalizing with the detected FLAG sequence. In naive tissue, antibody 6 performed the best, identifying specific bands in the brain cortex in Western analysis. These same antibodies produced a more diverse band profile in the vena cava, identifying 6 major proteins. In immunohistochemical experiments, the same C-terminus antibodies, with antibody 3 performing the best, detected the 5-HT_7_ receptor in rat veins. This deliberate work has given rise to at least three antibodies that can be used with good confidence in r5-HT_7_ transfected cells, two antibodies that can be used in immunohistochemical analyses of rat tissues and in Westerns of rat brain; we are less confident of the use of these same antibodies in rat veins.

**Supplementary Information:**

The online version contains supplementary material available at 10.1007/s00210-023-02482-w.

## Introduction

Serotonin (5-hydroxytryptamine or 5-HT) is a neurotransmitter/hormone that interacts with a large class of G protein coupled receptors (GPCRs) dedicated to one agonist. By last count, there were 14 receptor subtypes (Barnes et al. 2021). Of these, the most recently discovered is the 5-HT_7_ receptor. This receptor serves a myriad of functions including mood, depression, sleep and wakefulness, temperature regulation, gut function, immune cell regulation and, as we and others have discovered, blood pressure regulation (Blattner et al. 2019; Diaz et al. 2008; Gellynck et al. 2013; Kim and Khan 2014; Monti and Jantos 2014; Quinetero-Villegas and Valdes-Ferrer 2019).

Several lines of evidence support the ability of the 5-HT_7_ receptor to mediate a fall in blood pressure (hypotension) when infused into the rat (Diaz et al. 2008). These include the ability of the 5-HT _1A/7_ receptor agonist 5-carboxyamidotryptamine to reduce blood pressure; the 5-HT_7_ receptor antagonist SB269970 (Seitz et al. 2016, 2017, 2019) reducing 5-HT-induced hypotension; and the inability of 5-HT to cause a hypotension in the 5-HT_7_ receptor knockout (KO) rat (Demireva et al. 2019; Seitz et al. 2019). Our studies have narrowed down to a mechanism of dilation of the vasculature – both veins and small arteries/arterioles—as being mechanistically important for this hypotension (Seitz et al. 2017, 2021).

We have not, however, been able to demonstrate the sites of the 5-HT_7_ receptor protein itself. This is because of a lack of confidence in the many commercially available 5-HT_7_ receptor antibodies. Specificity of antibodies against GPCRs has posed considerable problems (Josti 2021; Michel et al. 2009; Tripathi et al. 2016). Validating where the 5-HT_7_ receptor is localized within a tissue is important in at least two ways. First, we could demonstrate that the 5-HT_7_ receptor protein is in those sites in which pharmacological evidence supports that they are (Watts et al. 2015, 2022; Seitz et al. 2021). Our evidence supports that 5-HT_7_ receptor mRNA is present in rat veins and skeletal muscle arterioles. Pharmacological experiments support that the 5-HT_7_ receptor mediates vessel relaxation in both sites (Watts et al. 2015, 2022; Seitz et al. 2021). Second, we could examine other tissues as additional sites of action as well as finding potential off target sites of activators of the 5-HT_7_ receptor.

We contacted 7TM Antibodies in Jena Germany to create a rat 5-HT_7_ receptor antibody. Here we present the work we have done independently and together to test the hypothesis that a specific rat 5-HT_7_ receptor antibody could be developed. We include experimental approaches that were successful (in formal text) and approaches that were unsuccessful (supplemental materials). We focused on two tissues with recognized functional 5-HT_7_ receptors: isolated rat veins (Watts et al. 2015, 2022) and the rat brain cortex (Fukuyama et al. 2022; Hrnjadovic et al. 2021; Labus et al. 2021; Lee et al. 2021; Liu et al. 2021; Solas et al. 2021). Our goal was to provide not only a useful antibody for this receptor but to also help our colleagues commiserate with the myriad of problems we ran into in development of this antibody.

## Methods and results

### Animal use at 7TM and MSU

Rabbits (ZIKA strain (Zimmermann Kaninchen, Germany), all female 3.5 month old) were used within an approved protocol within 7TM for generation of sera containing antibodies of interest. The Michigan State University (MSU) Institutional Animal Care and Use Committee approved all protocols used in this study at MSU (specific approval PROTO202100309). MSU is an Association for Assessment and Accreditation of Lab animal Care or AAALAC-accredited institution (A3955-01). Rats were used in accordance with the Guide for the Care and Use of Laboratory Animals (8th ed, 2011). Male Sprague Dawley rats were purchased from Charles River Laboratory (Kingston, NY, USA; RRID:RGD_10395233). Each n value represents data that came from one (1) animal. Rats were euthanized with pentobarbital (80 mg/kg, ip) and a bilateral pneumothorax created. Tissues of interest (brain, veins) were isolated, cleaned, flash frozen in liquid N_2_ and stored in a –80 °C freezer before use.

### Design of original three peptides as 5-HT_7_ receptor antigens at 7TM

**Fig. 1:**
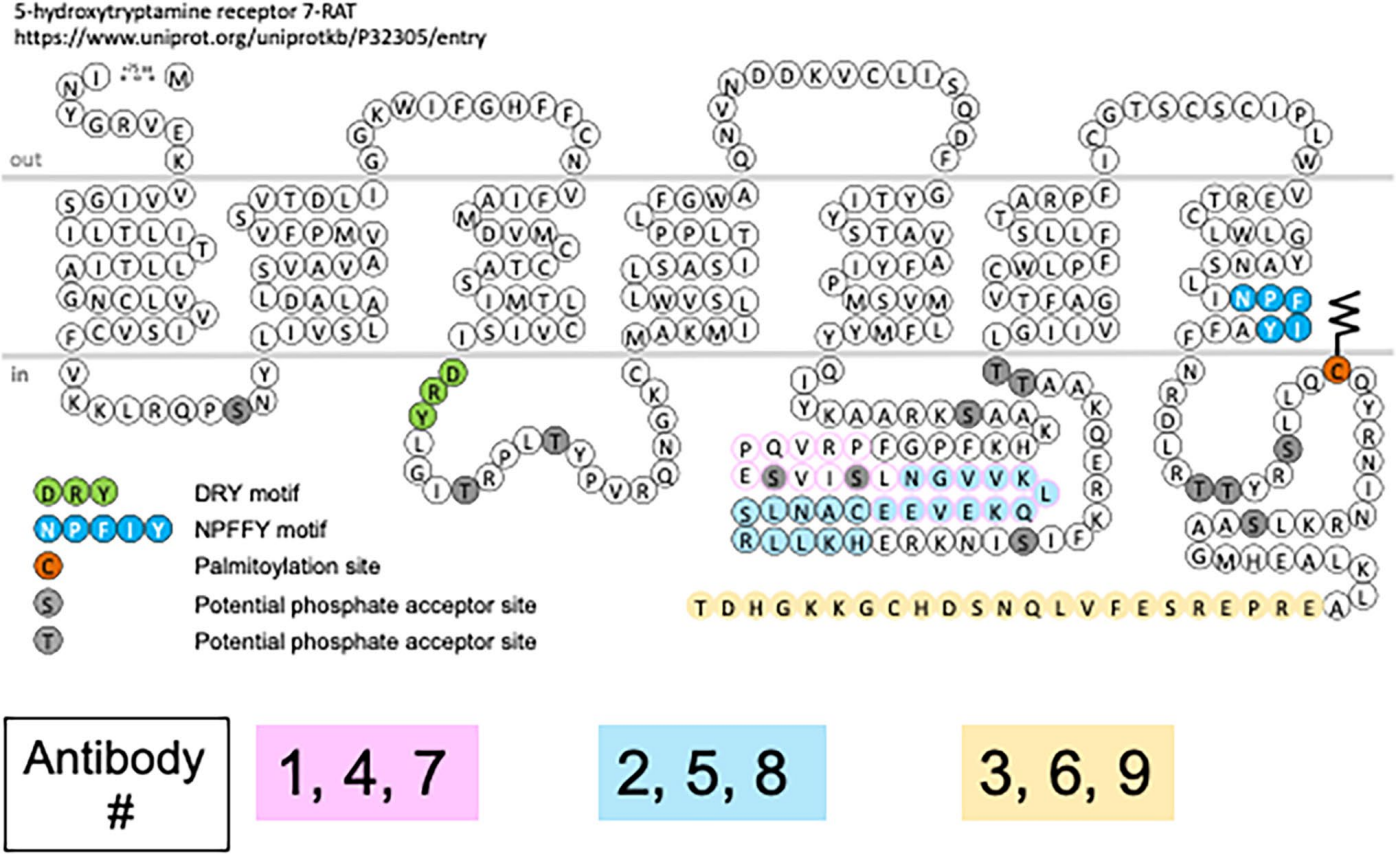
Snake plot of rat 5-HT_7_ receptor (r-5-HT_7_) with the peptide 1 (pink), peptide 2 (blue) and peptide 3 (yellow) epitopes marked within the snake plot. Plot was generated from data at https://ww.uniprot.org/uniprotkb/P32305/entry. Below the snake plot is the key for the antibodies raised against peptide 1 (antibody 1, 4 and 7 respectively), peptide 2 (antibody 2, 5 and 6 respectively) and peptide 3 (antibody 3, 6 and 9 respectively)

Figure 1 depicts the three (3) regions of the r5-HT_7_ receptor that were used to develop the 5-HT_7_ receptor antibodies. These epitopes were chosen because the N-terminus of GPCRs is often glycosylated, making it a difficult epitope. Both the third intracellular loop and the C-terminus of the r5-HT_7_ receptor are of significant size and, of GPCRs, are relatively unique for a given receptor. As such, all two epitopes from central part of the third intracellular loop and one epitope from the distal part of the C-terminus were chosen. Peptides were synthesized and coupled to keyhole limpet hemocyanin, and the conjugate was emulsified with Freund’s adjuvant. Rabbits were injected with one of these three antigenic sequences, and three rabbits were used for each sequence. Animals were injected at 4-week intervals, and serum was obtained 2 weeks after immunizations beginning with the second injection. Affinity purification of the r5-HT_7_ antisera was performed using the SulfoLink Kit (Pierce, Rockford, IL, USA, catalog # 44,999) as recommended by the manufacturer. In all subsequent experiments, affinity-purified antibodies were used.

### Transfection of HEK293T cells with r5-HT_7_ receptor expressing plasmid at MSU

Rat Htr7 (r5-HT_7_; P32305) cDNA/ORF clone (NovoPro Biosciences LTD, Shanghai, China; catalog #746,094–1) was purchased. This clone also contained a C-terminal FLAG sequence for tracking (vector pcDNA3.1-3xFLAG). This same plasmid was sent to 7TM (Jena, Germany) for independent cell transfection (below) in human embryonic kidney 293 or HEK293 cells.

For western blot analysis, approximately 1 × 10^6^ HEK293T cells (a kind gift from Dr. Richard Neubig’s laboratory at Michigan State University) were plated in a 60 mm dish (200,000 cells/ml, 5 ml media) and transfected with either 2 μg of rat 5HT_7_R or pcDNA3.1-Zeo (-) (Invitrogen/ThermoFisher, Waltham, MA, USA; catalog #V86520) as an empty vector control. At this time, cells were in Optimem (Gibco/ThermoFisher, Waltham, MA, USA; catalog #11,058–021) with Lipofectamine 2000 (Invitrogen/ThermoFisher; catalog # 11,668–019) for 4 h following the manufacturer’s instructions. After the 4-h incubation, media was changed to 10% fetal bovine serum (FBS; Gibco/ThermoFisher; catalog # 10,437–028) Dulbecoo’s Modified Eagle Medium (DMEM; Gibco/ThermoFisher; catalog # 11,995–065) with penicillin/streptomycin (Gibco/ThermoFisher; catalog # 15,140–122) and allowed to incubate overnight. Cells were then washed with phosphate buffered saline (PBS; Gibco/ThermoFisher; catalog # 10,010–023) and scraped into microcentrifuge tubes for further processing.

For immunocytochemical analysis, HEK293AD (adherent) cells were transfected using the same protocol as the HEK293T cells (gift from Dr. Richard Neubig’s laboratory at Michigan State University). After the overnight incubation with 10% FBS DMEM media, cells were trypsinized (Gibco/ThermoFisher; catalog # 25,300–054) and plated onto poly-D-lysine (Sigma Chemical Co, St. Louis, MO, USA; catalog # P7280) coated glass coverslips. Twenty-four hours later, the media was removed, cells were washed with PBS, and fixed with 4% paraformaldehyde (Biotium, Fremont, CA, USA; catalog # 22,023) for 10 min at room temperature. Of these plated cells, one 6 mm dish was kept for western blot analysis for verification of transfection.

### Westerns to confirm antibody potential at 7TM

#### Western analysis

Rat 5Htr7 cDNA/ORF Clone was transfected into HEK cells using the calcium phosphate precipitation method. Approximately 1.5 × 10^6^ cells were transfected with 20 μg of plasmid DNA. Cells were selected in the presence of 500 μg/ml G418 (Life Technologies, Eggenstein, Germany; catalog # 10,131,027). Wheat Germ Agglutinin (WGA) agarose bound beads (Vector Laboratories, Burlingame, CA, USA; catalog # AL-1023–5) were washed with ice-cold detergent buffer [50 mM Tris–HCl, pH 7.4, 150 mM NaCl, 5 mM EDTA, 1% Nonidet P-40, 0.5% sodium deoxycholate, 0.1% sodium dodecyl sulfate (SDS)], centrifuged briefly to collect at the bottom of the tube, and buffer removed. Supernatant from harvested cells was incubated with beads for 2 h at 4 °C with rotation. Samples were briefly centrifuged to collect beads at bottom of tube, supernatant aspirated, and discarded. Beads were washed 3 × 10 min with Tris Buffered Saline/1% Tween-20 (TBS-T) and the final wash aspirated and discarded. SDS-Sample Buffer (60 µl) was added to samples and incubated at 50 °C for 20 min. Samples were centrifuged at 14,000 × *g* for 5 min and 20 µL immediately loaded for electrophoresis on a 10% polyacrylamide gel, 130 V for 1 h. Protein was transferred (0.5 A, 25 V with Trans-Blot® Turbo system for 15 min) to polyvinylidene fluoride-FL (PVDF-FL) and membrane blocked in 5% nonfat milk for 3 h at 4 °C with gentle rocking. Primary antibody (each of the individual 9 developed antibodies) was added at 1:500 and membrane incubated overnight at 4 °C with gentle rocking. Primary antibody was removed, blot was washed 3 × 10 min with TBS-T and incubated with anti-rabbit-horse radish peroxidase (HRP)-coupled (Anti-rabbit IgG HRP linked, Cell Signalling, Danvers, MA, USA; catalog #7074) overnight at 4 °C with rocking. Secondary antibody was removed, blot was washed 3 × 10 min with TBS-T and incubated with HRP-substrate. Chemiluminescence was detected via X-ray film. Images were not modified from original procurement.

### Western protocol to test all 9 antibodies at MSU

#### Cell protocol

##### Transfected cell protocol

Approximately one million HEK293T transfected or non-transfected cells were added to 200 μL detergent buffer (pH 7.4 20 mM, NaCl 150 mM, EDTA 5 mM, Triton X-100 1%, glycerol 10%, sodium dodecyl sulfate 0.10%, phenylmethylsulfonylfluoride 0.2 mM, leupeptin 10 mg/mL, aprotinin 1 mg/mL), pipet-mixed 15X, then sonicated with a 6-s pulse at 10% amplitude. Samples were centrifuged at 10,000 rpm for 10 min at 4 °C and stored at − 80 °C. Protein concentration was determined using a bicinchoninic acid or BCA Kit (Millipore Sigma, St. Louis, MO, USA; catalog # BCA-1KT).

##### Western analysis

In some experiments, 500–2000 μg total protein was added per lane with the intent to test the concentration-dependence of the antibody signal. Acrylamide gels were either poured in lab into Criterion cassettes (Bio-Rad Laboratories, Hercules, CA, USA; catalog # 3,459,902, #3,459,903) or ordered as precast (Bio-Rad Laboratories; catalog # 5,678,044, # 5,678,045) and run under SDS-PAGE conditions at 120 V for ~ 2 h. Protein was transferred to a PVDF-FL membrane (Millipore Sigma; catalog # IPFL00010) at 100 V for 1 h and membrane air-dried. Total protein was determined using Revert Total Protein Stain (LI-COR, Lincoln, NE, USA; catalog #926–11,021) according to manufacturer’s protocol and scanned on the LI-COR Odyssey CLx 700 channel (LICOR RRID:SCR_013715). Membranes were rehydrated in methanol and Total Protein Stain removed using Revert Reversal Solution (LI-COR; catalog # 926–11,013) according to manufacturer’s protocol. Membranes were blocked in 5% nonfat milk (Bio-Rad Laboratories; catalog # 1,706,404) for 3 h at 4 °C. In some experiments, membranes were cut to create smaller individual blots for antibody testing. Blots were incubated with one of the 7TM antibodies at 1:500 dilution for 48 h at 4 °C. Primary antibody was removed, blots were washed 3 × 10 min with TBS-T, incubated with IRDye 800 goat anti-rabbit secondary antibody (1:1000; LI-COR; catalog # 926–32,211) for 1 h at 4 °C with rocking, washed 3 × 10 min with TBS-T, and scanned on the Odyssey CLx 800 channel to determine the ability of the 7TM antibodies to bind specific proteins in the homogenates. In some experiments, an antibody against the C-terminus FLAG protein sequence (Sigma-Aldrich, St. Louis, MO, USA; catalog #F3040) was added to blots previously exposed to 7TM antibodies, followed by addition of a LICOR IRDye 680 goat anti-mouse secondary antibody (LI-COR; catalog # 926–68,070) to detect colocalization of the 5-HT_7_ receptor signal with that of FLAG.

#### Naive rat tissue protocol

##### Protein lysate protocol

Previously frozen brain cortex (20–50 mg) and abdominal vena cava (7–15 mg) were added to Lysis Buffer [50 mM Tris–HCl, 150 mM NaCl, 5 mM EDTA, 1% Nonidet P-40, 0.5% Na-Deoxycholate, 0.1% SDS; 100 µL for tissues < 20 mg, 250 µL for tissues > 30 mg] in a 0.5 mL tube (Omni International, Kennesaw, GA, USA; catalog # 19–650) containing 0.17 g of 1.4 mm ceramic bead media (Omni International; catalog # 19–645-3). Samples were homogenized at 5.65 m/s, 30 s in an Omni Beadruptor 24 (Omni International) and mixed on a circular rotator for 30 min at 4 °C. Contents were transferred to 1.5 mL tubes and centrifuged at 15,000 rpm for 20 min at 4 °C. Supernatant was transferred to 1.5 mL tubes and stored at –80^o^ C. Protein concentration was determined using BCA Kit (Millipore Sigma, Burlingame, MA, USA; catalog # BCA-1KT).

##### Western analysis

Two hundred (200) µg brain cortex and abdominal vena cava protein were incubated with SDS Sample Buffer [62.5 mM Tris (pH 7.6), 2% SDS, 20% glycerol, 100 mM dithiothreitol, 0.005% bromophenol blue] for 10 min at 55 °C and electrophoresed on a Criterion 12% TGX Stain-Free gel (Bio-Rad Laboratories, Hercules, CA, USA; catalog # 5,678,044) at 120 V for ~ 1 h. Protein was transferred to a PVDF-FL membrane (Millipore Sigma; catalog # IPFL00010) at 100 V for 1 h and membrane air-dried, then cut in half between the molecular weight markers. Total protein on the right half was determined using Revert Total Protein Stain (LI-COR; catalog #926–11,021) according to manufacturer’s protocol and scanned on the LI-COR Odyssey CLx 700 channel; the left side was unstained. Membranes were rehydrated in methanol and Total Protein Stain removed using Revert Reversal Solution (LI-COR; catalog # 926–11,013) according to manufacturer’s protocol. Membranes were blocked in 5% milk for 3 h at 4 °C, then incubated with a 1:1000 dilution of the 7TM antibody 6 for 15 h at 4 °C with rocking. Primary antibody was removed, blots were washed 3 × 10 min with TBS-T, incubated with IRDye 680 donkey antirabbit secondary antibody (1:5000; LI-COR; catalog # 926–68,073) for 1 h at 4 °C with rocking, washed 3 × 10 min with TBS-T, and scanned on the Odyssey CLx 700 channel.

### Immunocytochemistry/immunohistochemistry protocol at MSU to test all 9 antibodies

#### Immunocytochemistry protocol

Transfected HEK293AD cells fixed (HOW) on 18 mm glass coverslips were placed in 12-well Corning plates (Corning, Durham, NC, USA; catalog #3513) and washed with PBS (Sigma-Aldrich; catalog # D8537), then incubated for 10 min at room temperature with PBS + 0.25% Triton X100 (Research Products International, Mt. Prospect, IL, USA; catalog # 111,036). Coverslips were blocked in normal goat serum (Vector Laboratories, Newark, CA, USA; catalog # S-1000) for 1 h at room temperature, then incubated with one of the 7TM primary antibodies (1:500) and FLAG antibody (1:500, Abcam, Cambridge, UK; catalog # 117,495) overnight at 4 °C. The following day, coverslips were washed 3X with PBS and incubated with AlexaFluor488 goat anti-rabbit antibody (1:1000, Invitrogen, Waltham, MA, USA; catalog # A11008) for 1 h at room temperature. Coverslips were washed 3X with PBS and mounted on glass slides with ProLong Gold with DAPI (Vector Laboratories; catalog # H-1500). Slides were dried and imaged on a Nikon Eclipse Ti2-E Inverted Motorized Research Microscope with Perfect Focus System 4 and NIS-Elements AR Software (AI algorithms for optimal signal/noise imaging; 2D/3D deconvolution). Images shown are at 40 × magnification with exposure time of 50 ms for fluorescein isothiocyanate (FITC) and 400 ms for tetramethylrhodamine (TRITC) fluorophore channels.

#### Immunohistochemistry protocol

Tissues were fixed in formalin (10%) before being cut as slides from paraffin blocks by the MSU Investigative Histopathology services*.* Sections (8 micron thick) were placed on glass slides. Paraffin embedded sections of rat portal vein were dewaxed [3 min each] at room temperature as follows: incubation in Histochoice (VWR; catalog # H103-4L), 2X; isopropanol, 4X; dH_2_O, 2X)]. Slides were then antigen retrieved [Antigen Unmasking Solution, Vector Labs; catalog # H-3301) for 30 s at full power in the microwave. Sections were incubated with blocking serum (Vector Labs; catalog # S-1000) for 1 h at room temperature in a humidified chamber, then incubated overnight at 4 °C in a humidified chamber with one of the 7TM antibodies (1:100) or blocking serum (negative control).

The next day, sections were washed 3X with PBS (Millipore Sigma; catalog # D-8537) and incubated with AlexaFluor 488 goat anti-rabbit secondary antibody (1:500; Invitrogen; catalog # A11008) for 1 h at room temperature in a humidified chamber, then washed 3 × with PBS. Nuclei were identified using Vectashield Hardset Antifade Mounting Medium with DAPI (Vector Labs; catalog # H-1550) and coverslips were placed. Slides were dried completely and imaged on an inverted Nikon Eclipse TE2000 microscope (RRID:SCR_021068) with a Nikon Digital Sight DS-Qil camera and Nikon NIS Elements BR 4.6 software (RRID:SCR_014329). Images shown were taken at 20X magnification with an exposure time of 500 ms for the FITC fluorophore.

### Data analyses and image handling

Western images were developed using the Licor Odyssey (Image Studio *5.2.5*). For immunocytochemistry/immunohistochemistry, images were taken on a Nikon TE2000 inverted microscope using a Nikon Digital Sight DS-Qil camera and Nikon NIS Elements BR 4.6 software. Images of any type were brightened or contrasted as a whole, never in part. In Westerns, species specific far red secondaries were used to visualize signals for the FLAG tag (green) and r5-HT7 receptor (red) on the LICOR Odyssey. For imaging of cells or tissues, *FITC was the fluorophore used to track the r5-HT*_*7*_* while TRITC tracked the FLAG tag*. For immunocytochemistry, some cellular images were enlarged from original images to more closely identify cellular staining and demonstrate TRITC signaling that was difficult to observe with the strong FITC (r5-HT_7_) signal.

## Results

### Generation of test antibodies against the 5-HT_7_ receptor: work by 7TM

**Fig. 2:**
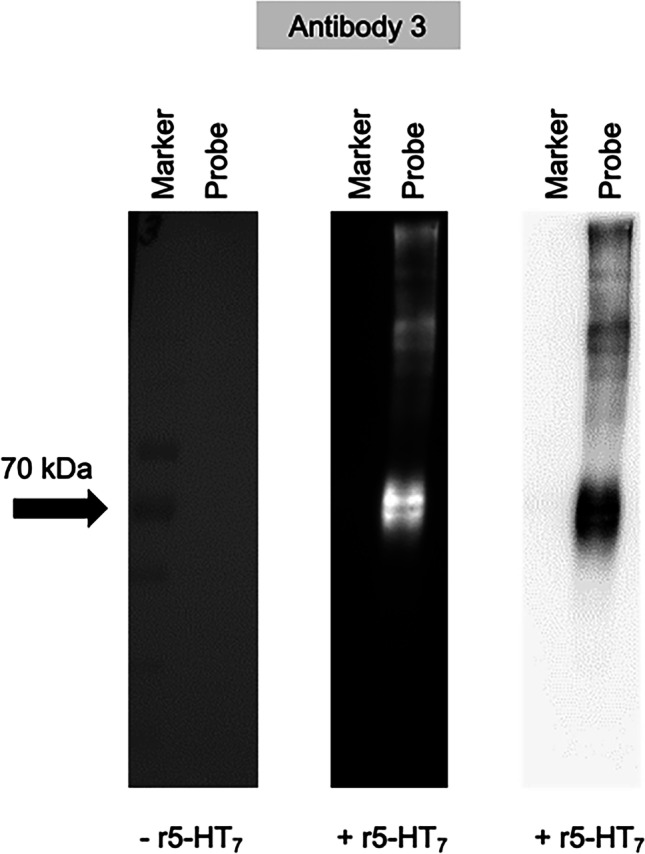
Western analysis of HEK293T cells non-transfected (-r5-HT_7_) and transfected (+ r5-HT_7_) when run on standard SDS polyacrylamide gel and probed with antibody 3 directed toward peptide 3. Representative of all nine antibodies generated by 7TM. Far left panel shows results in cell homogenate -r5-HT_7_. Mid and far right panel demonstrate ECL staining, both in inverted gray scale (mid) and gray scale (far right)

Three antibodies against the three different antigens depicted in Fig. 1 and listed in Table 1 were generated. Three separate rabbits each generated an antibody against these three epitopes. Antibodies generated against peptide 1 (proximal 3^rd^ intracellular loop; aa 282–305) are antibodies 1, 3 and 7. Antibodies against peptide 2 (more distal 3^rd^ intracellular loop; aa 293–314) are antibodies 2, 5 and 8. Finally, those antibodies against peptide 3 (far carboxy terminus; aa 426–448) are antibodies 3, 6 and 9. Using WGA beads to capture and concentrate the highly glycosylated GPCR and using antibody 3, 7TM identified an ~ 75 kDa band, with bands of higher molecular weight unidentified (Fig. 2). The image in Fig. 2 is an exemplar of results for all nine antibodies. These positive results required testing of all nine antibodies in the following experiments.

**Table 1:** Listing of the nine (9) antibodies created against the r5-HT_7_ receptor. Nomenclature of the nine (9) antibodies generated by 7TM. First column is antibody number used throughout study; second column is the peptide antigen as a linear amino acid sequence and the amino acid (aa) residues of the r5-HT7 receptor this represents, and third column demonstrates the three peptide antigen sequences were tested in three different rabbits

*n*		
Antibody Number	Peptide Sequence	Rabbit/ Batch #
1	PRVQPESVISLNGVVKLQKEVEE (r5-HT_7_ aa 282–305)	0174–1
2	NGVVKLQKEVEE(Abu)ANLSRLLKH (r5-HT_7_ aa 293–314)	0174–2
3	ERPERSEFVLQNSDH(Abu)GKKGHDT (r5-HT_7_ aa 426–448)	0174–3
4	PRVQPESVISLNGVVKLQKEVEE	0175–1
5	NGVVKLQKEVEE(Abu)ANLSRLLKH	0175–2
6	ERPERSEFVLQNSDH(Abu)GKKGHDT	0175–3
7	PRVQPESVISLNGVVKLQKEVEE	0176–1
8	NGVVKLQKEVEE(Abu)ANLSRLLKH	0176–2
9	ERPERSEFVLQNSDH(Abu)GKKGHDT	0176–3

### Antibodies against C terminus antigen identified r5-HT_7_ receptor at MSU

**Fig. 3:**
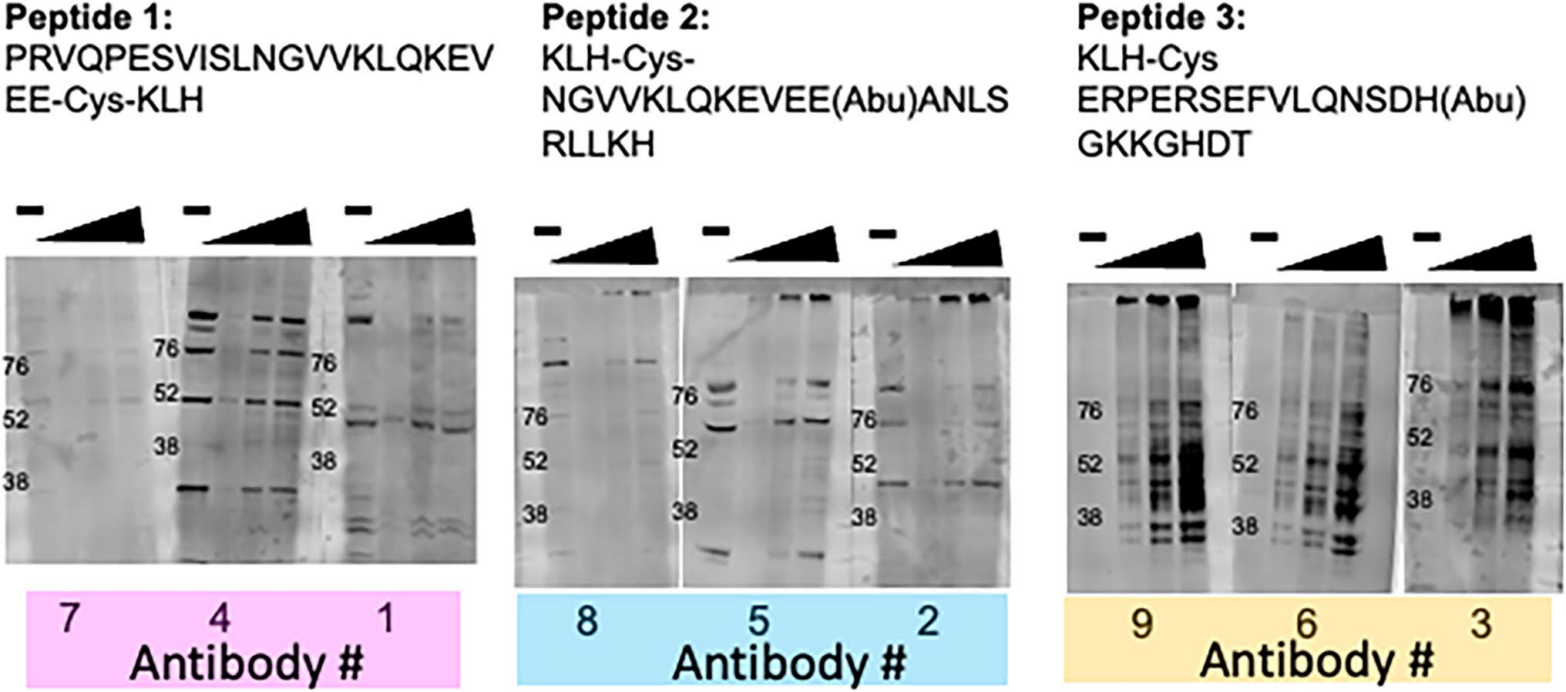
Westerns of all nine antibodies run on homogenates of HEK293T cells that were non-transfected (-) of transfected ( +) with the r5-HT_7_ plasmid. Miniblots are grouped by peptide antigen used, from peptide 1 [antibodies 7, 4, and 1] to peptide 2 [antibodies 8, 5, and 2] to peptide 3 [antibodies 9, 6, and 3]. Wedge indicates increasing amounts of total protein loaded in each lane

Figure 3 groups the nine antibodies by the antigen (peptide 1, 2 or 3) used to develop the antibody. In each mini-blot, one lane was loaded with a high concentration of protein from cells that were not transfected with the r5-HT_7_ receptor plasmid. The next three lanes were loaded with a low (500 μg), mid (1,000 μg) and high (2,000 μg) amount of total protein from cells that were transfected with the r5-HT_7_ receptor protein. Antibodies against peptide 1 and peptide 2 did not show specificity between transfected *vs* non-transfected cells. This was evidenced by bands being present in the lane with homogenates of non-transfected cells. Moreover, there was no quantitative increase in signal/banding pattern with increasing homogenate protein concentration (Fig. 3). By contrast, antibodies against peptide 3 (3, 6, and 9), directed against the far C-terminus of the rat 5-HT_7_ receptor, discriminated between homogenates without and with the transfected receptor. Moreover, as the total protein of the homogenate increased, the signal for multiple bands, ranging from over 100 to just above 36 kDa in mass, increased accordingly. These proteins were largely similar but not identical between the three antibodies raised against peptide 3 (Fig. 3, far right). Description of unsuccessful modifications around the Western protocol can be found in supplemental materials. We next determined whether the several bands detected by the 3, 6, or 9 antibody reflected the 5-HT_7_ receptor. Further results using antibodies against peptides 1 and 2 are shared in supplemental material.

### Multiple bands were detected by the antibody directed against Peptide 3 of the r5-HT_7_ receptor

**Fig. 4:**
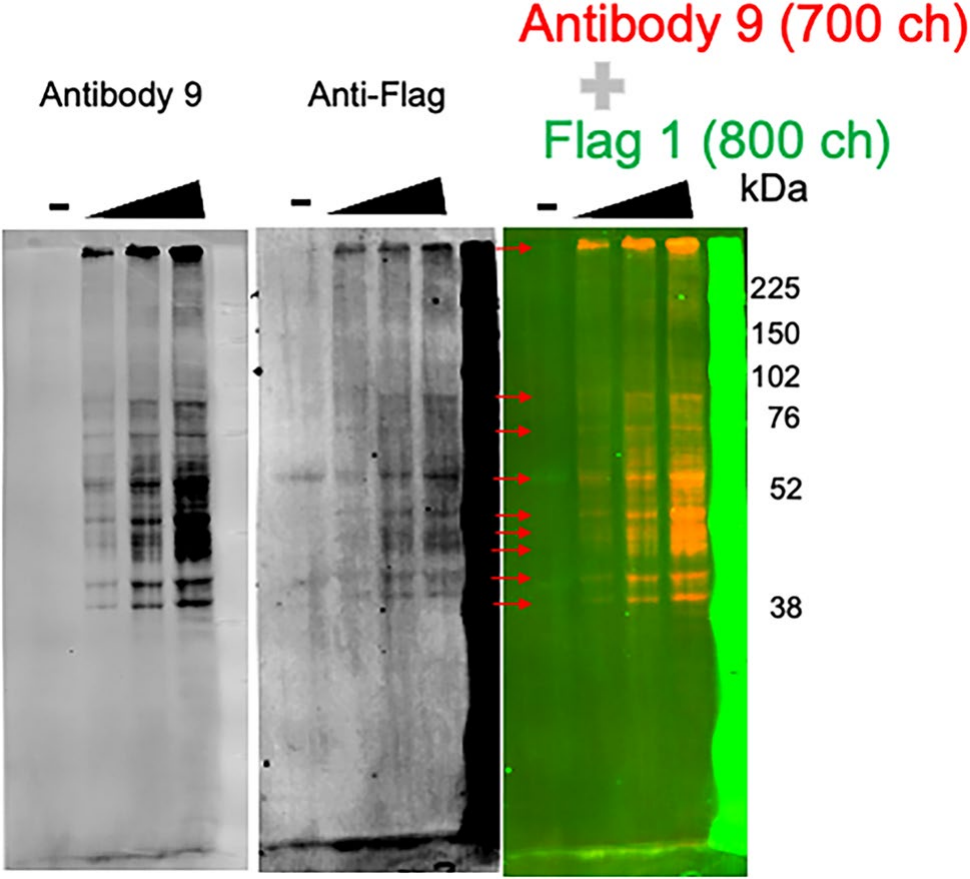
Representative Western of colocalization of the C–terminal FLAG epitope and r5-HT_7_ receptor epitope in homogenates of HEK293T cells that were non-transfected (-) or transfected ( +) with the r5-HT_7_ plasmid. Wedge indicates increasing amount of total protein loaded in each lane. Far left panel shows individual western using antibody 9 (developed in 700 channel of LICOR Odyssey), middle panel shows the FLAG signal alone (developed in 800 channel), and far right shows color colocalization of the two signals (yellow-orange) with molecular weight markers to the right. Red arrows on far left panel left border point out FLAG/r5-HT_7_ co-labelled bands

For the antibody 9, westerns of cells transfected with or without the r5-HT_7_ receptor were transferred to immunoblot that was exposed to both the antibody 9 and a C-terminus anti-FLAG antibody. Figure 4 depicts multiple bands being recognized as the 5-HT_7_ receptor, validated by colocalization with a FLAG antibody. The primary bands were between 38 and 59 kDa; these sizes are consistent with GPCR molecular weights. However, there are likely post translational modifications or truncations that cause the different apparent MWs of peptides that are FLAG-labelled. For example, bands of a smaller molecular weight than 48 kDa were observed. Similarly, protein was left unresolved at the stacker/running gel interface that also stained for the FLAG antibody. The faint green bands in the negative line are likely due to spillover from the + lane; in other experiments, this lane is empty. This particular image was chosen because it best demonstrates colocalization of the FLAG signal (FITC here) We cannot rule out that processing of the r5-HT_7_ receptor in these cells resulted in proteins of these unexpected molecular weights.

### Antibodies against peptide 3 detected the 5-HT_7_ receptor in rat tissues

**Fig. 5:**
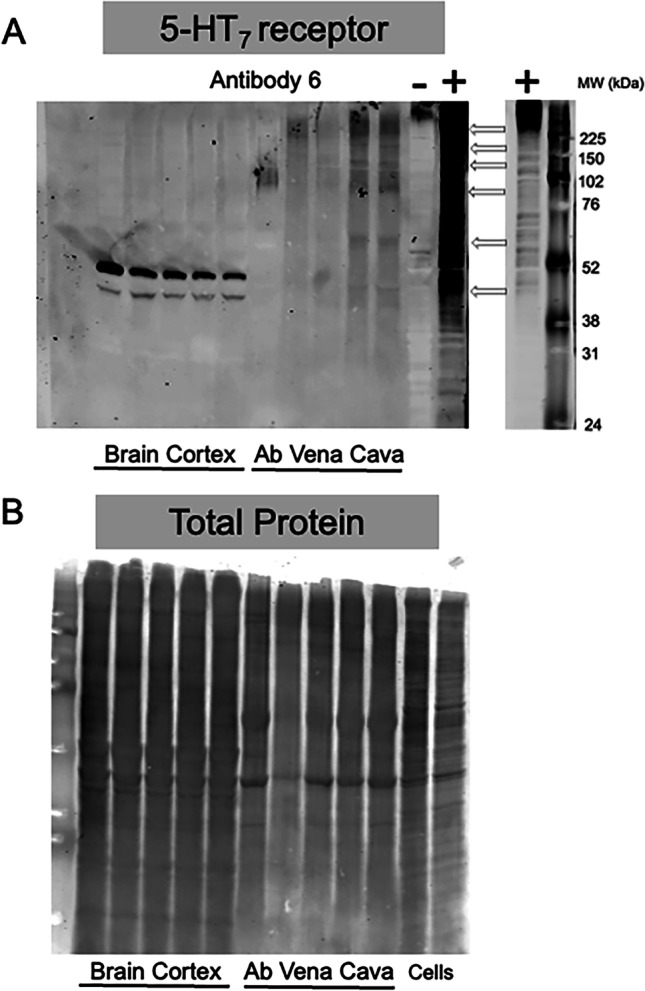
Detection of the 5-HT_7_ receptor protein in rat brain cortex and abdominal vena cava (**A**). **B** depicts that total protein stain of a blot running homogenates from the same cortex and abdominal vena cava in tandem with that exposed to antibody 6 (against peptide 3). Supplemental materials describe how the LI-COR Total Protein Stain interfered with development of blots with an antibody; the blot exposed to the r5-HT_7_ receptor antibody never received the Total Protein Stain. Arrows registers the most dominant bands recognized in the cortex or Ab Vena Cava to r5-HT_7_ expressing cells and the molecular weight markers on the far-right hand side

We next tested antibody 6 (raised against peptide 3) in its ability to detect naïve rat 5-HT_7_ receptor. Protein (200 μg total) of homogenates of brain cortex and abdominal vena cava were run from five different male Sprague Dawley rats, the protein of which is present in each individual lane. Detected bands were marked with a thin colored line drawn through them and to the corresponding MW markers on far-right hand side of Fig. 5A. A strong band at approximately 50 kDa was observed in the brain cortex (Fig. 5A). Bands in the vena cava were less clear. In the vena cava, positive bands were present at 6 different molecular weights (~ 42, 60, 80, 140, 180, and 230 kDa), none of which were similar to the primary band observed in the brain cortex in either shape or size. Importantly, the total protein stain for this blot demonstrated consistent loading of protein, including the abdominal vena cava samples (Fig. 5B). Total protein was generated independently given that total protein stain for visualization on the LICOR created artifactual bands at molecular weights that could be interpreted as a positive signal (supplemental material text).

### Peptide 3 antibodies successfully detected 5-HT_7_ receptor immunocytochemically

**Fig. 6:**
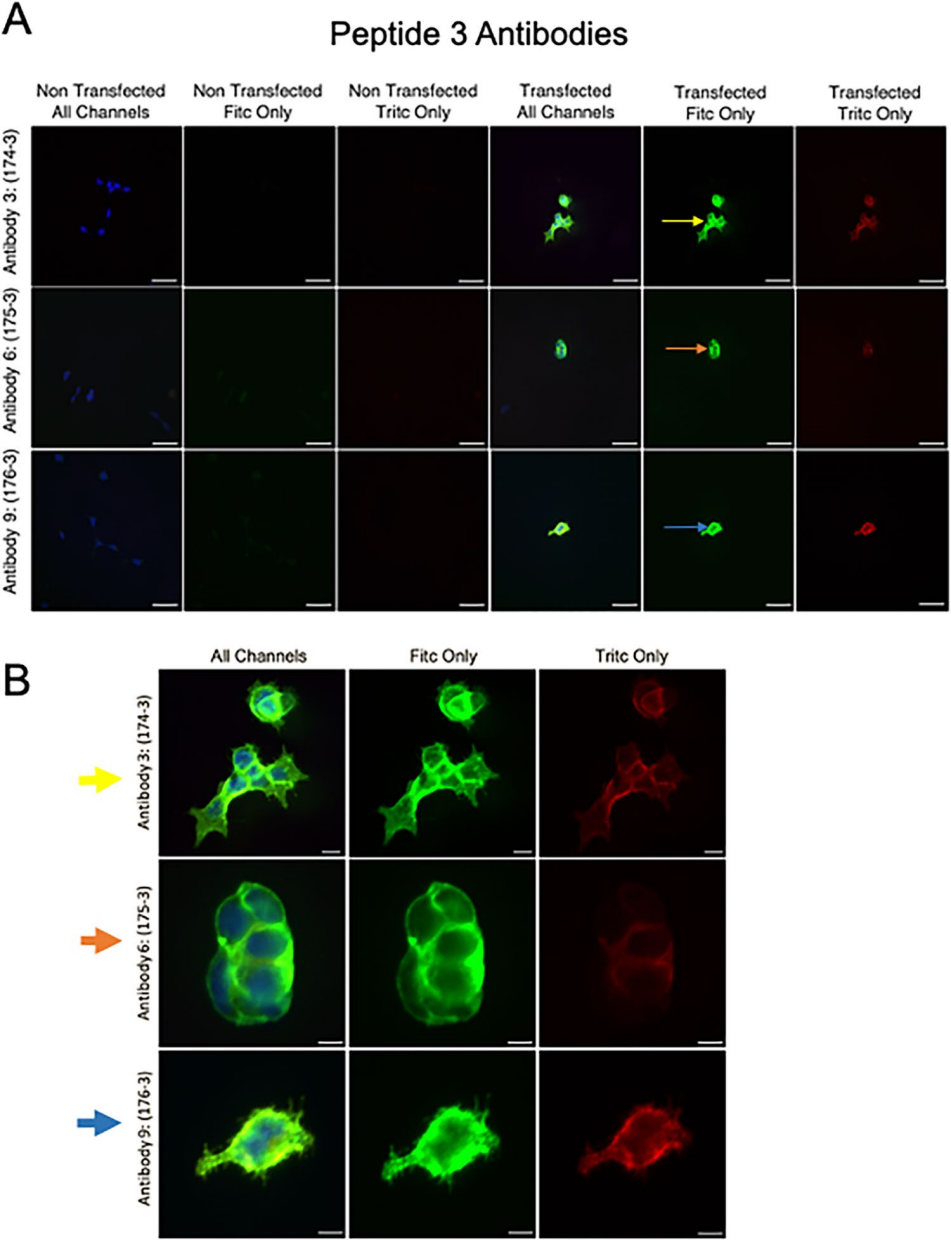
Immunocytochemical results against all three antibodies against peptide 3. **A** Parallel experiments were done in cells that were both non-transfected (-) or transfected ( +) with the r5-HT_7_ plasmid. FITC channel visualizes the 5-HT_7_ receptor staining while TRITC is staining for the FLAG epitope in the same cells. “All channels” is the overlay of the FITC + TRITC channel. **B** Depiction of a greater magnification of the positive signal observed in **A**, matching panels in **A** and **B** through colored arrow at the left hand side. Scale bar bottom right = 50 µm (**A**) and 10 µm (**B**)

Figure 6 shares exemplar experiments done to test whether antibodies against peptide 3 could be successful in immunocytochemical experiments. Here we used the HEK293AD cells both transfected and non-transfected with the r5-HT_7_ receptor plasmid. In addition, an antibody against the same C-terminal FLAG (as used in Westerns above) was used concurrently to colocalize with fluorescence from the secondary that detected the 5-HT_7_ receptor. All three rabbit antibodies derived against Peptide 3 (3, 6, and 9) detected transfection dependent signal and colocalization, though immediately less clear in this panel, of the 5-HT_7_ receptor signal with FLAG (right three panes of Fig. 6A); this was absent in the non-transfected cells. Figure 6B shares magnification of the same cells identified within Fig. 6A and demonstrates significant signal along the cell membrane. The far panel of Fig. 6B is a brightened TRITC image to demonstrate that this FLAG based signal was present but dominated by the FITC signal in the overlay. Results for antibodies against peptide 1 and peptide 2 can be found in supplemental Fig. 1.

### Peptide 3 antibodies detect 5-HT_7_ receptor protein in isolated rat vein in immunohistochemical experiments

**Fig. 7:**
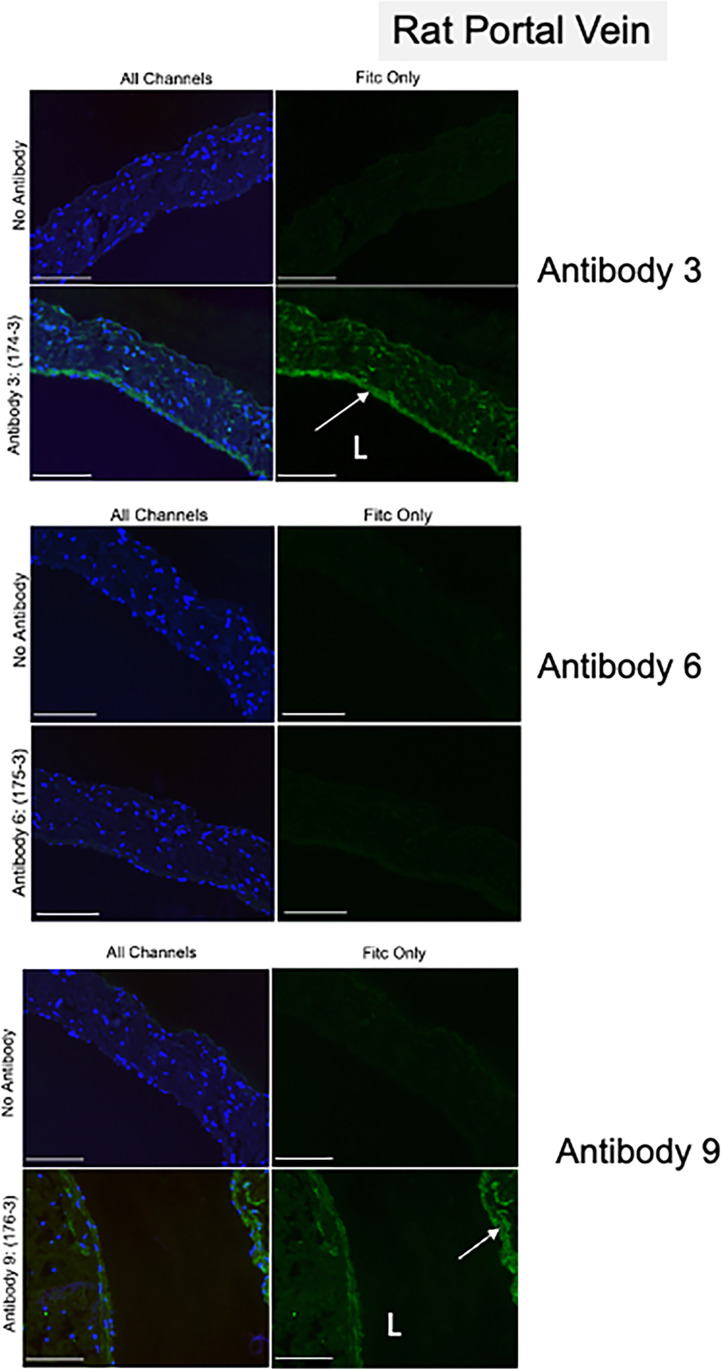
Immunohistochemical results in isolated rat portal vein using all three antibodies against peptide 3 (antibody 3, 6, and 9). No primary = primary left out of reaction. All channels = r5-HT_7_ receptor signal + DAPI (blue). Representative of three-four different male Sprague Dawley rats. White arrows = regions of interest, L = lumen of vein. Scale bar bottom right 100 µm

Figure 7 shares immunohistochemical experiments using antibodies only against peptide 3 (antibody 3, 6, and 9). Antibody 3 was clearly superior in detecting the 5-HT_7_ receptor, expressed in the only smooth muscle cell layer present in the portal vein (Fig. 7; supplemental Fig. 2). Similar staining was less brilliant with antibody 9, and could not be detected with antibody 6. Thus, immunohistochemical ranking places from best to worst antibody 3 > 9 > 6.

## Discussion

This manuscript shares work done over two years in a collaboration between MSU and 7TM to arrive at an antibody against the 5-HT_7_ receptor that was specific and selective against the rat 5-HT_7_ receptor. There have been long standing concerns as to the ability of antibodies against GPCRs to serve with both selectivity and specificity. This is understandable given the overall structural homology of GPCRs, but creates a challenge for the experimental scientist.

The 5-HT_7_ receptor has been implicated in numerous physiological processes and is under appreciated. These include the physiological functions of mood (Fukuyama et al. 2022), nociception (Nascimento et al. 2021), gastrointestinal function, learning (Hrnjadovic et al. 2021), memory (Labus et al. 2021), and circadian rhythm (Liu et al. 2021), to name a few. Similarly, this receptor is proposed to contribute to the pathophysiology of many diseases that include Alzheimer’s (Solas et al. 2021), neurodevelopmental disorders (Lee et al. 2021), and prostate cancer (Cinar et al. 2021). Our specific focus has been on the ability of the 5-HT_7_ receptor to mediate the hypotensive effects of both acutely and chronically infused 5-HT (Diaz et al. 2008). Our research would be stronger if the location/site of the 5-HT_7_ receptor, specifically in the vasculature, could be identified. It is for this reason we reached out to 7TM to carry out this collaborative work.

### Antibodies directed towards far C-terminus were successful in both Westerns and ICC/IHC

We carried out deliberate, step-wise experiments with all nine antibodies created by 7TM, using the separate three antibodies each developed against each of the three epitopes. Multiple independent investigators arrived at antibodies 3, 6, and 9 as those which identified the rat 5-HT_7_ receptor in a specific and protein-dependent manner. We conclude this by comparing the positive results from the HEK293T cells transfected with the r5-HT_7_ receptor which were taken through Western analyses and immunocytochemistry, as well as the lack of ability of the other antibodies (1, 4, 7 and 2, 5, 8) to demonstrate specificity and selectivity of signal. This comprised a rigorous approach in that more than two individuals came to the same conclusion that these antibodies against the far C-terminus (aa 426–446) of the rat 5-HT_7_ receptor were those with the greatest possibility for being developed into useful laboratory tools. These transfected cells are, however, the idealized tissue to study as they are purposefully significantly enriched in the intended target. The real test came in examining if these same antibodies could detect the 5-HT_7_ receptor in naïve rat tissues.

### Does success in transfected cell translate to native tissue?

This work was done to test, ultimately, if these antibodies could be successfully used in tissues from the rat expressing native, endogenous 5-HT_7_ receptors. The two (2) test tissues, veins and the cortex, were used with purpose. As stated above, central 5-HT_7_ receptors are important for numerous physiological endpoints. In the cardiovascular system, 5-HT causes 5-HT_7_ receptor-dependent relaxation in the isolated veins (Watts et al. 2015) and mRNA for the 5-HT_7_ receptor can be reproducibly measured in isolated rat veins (Watts et al. 2022). Accordingly, immunohistochemistry of the portal vein demonstrates that antibody 3 has the ability to bind to the 5-HT_7_ receptor in the single smooth muscle layer that exists in this tissue. This was modestly apparent with antibody 9 and not at all with antibody 6. We do not know why these two C-terminus directed antibodies, similar in their antigenic derivation, were less effective than antibody 3. We have published on use of a 5-HT_7_ receptor antibody in isolated rat tissue (Watts et al. 2015). However, the antibodies used in that previous paper did not go through the high level of rigor presented here. We have long been concerned as to our ability to reliably detect the r5-HT_7_ protein. Thus, we pushed to do the present study to have a tool in an antibody we trusted. Our findings clearly support use of the antibodies directed towards the far C-terminus. Why antibody 6 worked best in Westerns while antibody 3 worked best in immunohistochemistry is a frustrating mystery we have not solved. This inability to use an antibody in all antibody-based assays is well recognized, evidenced in the datasheets on antibodies sold by multiple companies.

The r5-HT_7_ receptor appears to be processed differently when placed in different contexts. From the HEK293T cells, to the cortex, and to the vein, the banding patterns for the 5-HT_7_ receptor were not the same. Western analyses defined a prominent single band in the brain homogenates; this was not the case in homogenates of vena cava. The band most commonly identified in Westerns was ~ 50 kDa, consistent with the molecular weight of many other GPCRs. However, there were several more bands that were both smaller and larger in molecular weight than 50 kDa in the transfected cells and in the vena cava. These differences raise the idea that this receptor is heavily processed by the cell in which it is in, and not processed identically in cells. For example, post translational modification of the 5-HT receptor is supported by several studies. Specifically, the 5-HT_7_ receptor undergoes N-glycoslyation (Gellynck et al. 2012) and C-terminal palmitoylation (Kvachina et al. 2009). We have not tested whether a glycosylase would narrow the width of the bands observed. In addition, we cannot exclude the possibility that the r5-HT_7_ receptors dimerizes or forms oligomers with other proteins (including other GPCRs), the complexes of which survive the processing for Western analyses. Along this same line, we also did not actively take into account that the 5-HT_7_ receptor has splice variants given that these splice variants result in proteins largely similar in their pharmacological and signalling functioning. As such, we made an assumption that the developed antibodies would have similar affinities from all receptors expressed from splice variants. Our Western analyses supports.

### Limitations

These antibodies were raised with a specific focus on the rat 5-HT_7_ receptor. We did not set out, nor did we expect, to create the perfect antibody that would work faithfully in Westerns and immunohisto/chemical analyses. We arrived at an antibody that can be used in immunohistochemistry and Westerns, at least in brain tissue. Here, we recognize limitations of our work. First, we do not know if they would successfully identify the 5-HT_7_ receptor in other species. Second, we have not successfully tested these antibodies (#1-#9) in any other antibody-based detection system such as immunoprecipitation, flow cytometry, etc. We also have not investigated other means of fixing or using fresh frozen tissue. Now that we have working antibodies in hand, this can be our next step to determine if detection could be improved by changes in fixation or embedding. It was imperative that we first had antibodies that even had a hope of being used successful, and this we have verified.

Third, we have created a functional 5-HT_7_ receptor KO rat (Demireva et al. 2019). It would be sensitble to test the described antibodies in tissues (veins, cortex) from the KO *vs* the WT. However, though the 5-HT_7_ receptor is not functional in isolated veins of the KO *vs* WT 5-HT_7_ receptor rat, tissue from the KO continues to express mRNA recognized by 5-HT_7_ primers. We simply do not know if the 5-HT_7_ receptor is expressed but not functional in these rats. As such, the 5-HT_7_ receptor KO rat does not provide a good control for us to use. In fact, one of the reason the antibodies in this study were developed is to help us determine if the 5-HT_7_ receptor protein can be detected in the KO. Finally, we have not sequenced the bands of interest in our Westerns of naïve tissue. We have had continual difficulty with this process, being unable to have sufficient signal to rise above the noise that accompanies mass spectrophotometric determination of protein.

## Conclusions

We provide a methodological and deliberate study of creating and testing an antibody for detection of the rat 5-HT_7_ receptor.

## Supplementary Information

Below is the link to the electronic supplementary material.

Supplementary file1 (DOCX 22 kb)

Supplementary file2 (PDF 1128 kb)

## Data Availability

Original data will be made available upon reasonable request.
