# Supplementary material for: Development of a 5-HT7 receptor antibody for the rat: the good, the bad, and the ugly
Source: Naunyn Schmiedebergs Arch Pharmacol. 2023 Apr 18;396(10):2599–611. doi: 10.1007/s00210-023-02482-w (PMC10497691; doi:10.1007/s00210-023-02482-w)
Supplement: Supplementary file 1 — Supplementary file1 (DOCX 22 kb) [file 210_2023_2482_MOESM1_ESM.docx]

**Supplementary Information for:**

**Development of a validated 5-HT_7_ receptor antibody for the rat: the good, the bad and the ugly**

^1^Janice M. Thompson, ^1^Will Tragge, ^1^Emma D. Flood, ^2,3^Stefan Schulz, ^1^Erika Lisabeth and ^1^Stephanie W. Watts

^1^Department of Pharmacology and Toxicology, Michigan State University, East Lansing, MI 48824-1317

^2^Department of Pharmacology and Toxicology, Jena University Hospital, 07747 Jena, Germany

^3^7TM Antibodies, 07745 Jena, Germany

**Correspondence to:**

Stephanie W Watts

1355 Bogue Street, Rm B445

Department of Pharmacology and Toxicology

Michigan State University

East Lansing, MI 48824-1317

E-mail: [wattss@msu.edu](mailto:wattss@msu.edu)

Telephone: 517 353 3724

ORCID: 0000-0002-9653-6357

**Supplemental Materials**

Below is a summary of western optimization steps attempted with the nine 7TM antibodies.

Protein Concentration: Initial westerns were run using 200 ug of (-) and (+) 5-HT7 transfected HEK cells. This did not yield sufficient signal with the 9 antibodies to determine with certainty whether specific binding had occurred (data not shown). Concentrations were then raised to the 500-2,000 µg shown in Figure 3, resulting in strong banding patterns for all antibodies, including a concentration dependent increase in signal with antibodies 3, 6, and 9.

Lysis Buffer: To increase the potential for isolating a specific target band for the r5-HT_7_ receptor, Pierce GPCR Extraction and Stabilization Reagent, a buffer specific for isolating GPCR proteins, was used (ThermoScientific; cat. #A43436). No discernable differences or improvements were noted between this buffer and the detergent buffer (see Methods section). Following consultation with Stefan Schultz, 7TM Lysis Buffer (see Methods section) was used for all subsequent isolations.

Sonication: During initial protein isolations, cells were sonicated with a 6-second pulse at 10% amplitude. This step was replaced with the Beadruptor protocol used on tissues (see Methods section), allowing samples to be processed together while retaining signal consistency.

Temperature: During initial experiments, protein homogenates were boiled prior to electrophoresis, resulting in significant sample retention in the wells that did not run through the separating portion of the gel. This boiling step was replaced by sample incubation at 55^o^C which successfully eliminated this issue.

Secondary Antibodies: To eliminate non-specific bands, we investigated if nonspecific secondary attachment to protein occurred. Different species (goat and donkey) and channel (700 and 800) secondaries showed the same signal pattern. However, IRDye 680 donkey anti-rabbit was found to give the best signal to noise ratio.

Total Protein Stain Reversal: Carryover signal from Total Protein Stain (see Methods) was still visible on the 700 channel of the Odyssey CLx following Reversal step. When using the 700 channel for target band analysis, incomplete reversal left non-target band interference in primary antibody signal.

Immunoprecipitation with Protein A/G beads – nonspecific enrichment: Due to nonspecific bands seen across all antibodies tested, immunoprecipitation with protein A/G beads was done to potentially provide a more receptor specific signal. To a pellet containing one million (-) or (+) 5-HT_7_ HEK293T cells, 100 μL of GPCR buffer was added, the contents vortexed briefly to combine, then mixed on a circular rotator for 30 minutes at 4^o^C. Samples were centrifuged at 1500 rpm for 30 min at 4^o^C, supernatant transferred to new microcentrifuge tubes and protein concentration determined using BCA Kit (Millipore Sigma). Two hundred (200) µg of protein was incubated with 2 µg of 7TM primary antibody in PBS at a final volume of 2 mL [Millipore Sigma; cat. # D-8537] for 2 hours at room temp with gentle mixing four times over the incubation period. Thirty (30) µL Pierce Protein A/G Magnetic beads (ThermoScientific; cat. # 88802) were added and incubated overnight at 4^o^C with circular rotation. Samples were washed 3 times in 2 mL PBS and 35 µL Loading Buffer added. Samples were boiled for 5 minutes to elute the protein from the beads and samples were loaded onto the gel for electrophoresis as described in Methods. While the IP reduced some of the spurious signals in the lanes, there was a clear band in both cells with and without the receptor and the boiling step resulted in protein retention in the wells, as indicated in the Western Optimization section above. The boiling step was replaced by elution with Pierce IGG Elution Buffer, pH 2 (Fisher Scientific; cat. # PI21028) in subsequent experiments, reducing sample retention in the wells, however, significant non-specific bands were once again visualized.

As in Western experiments, antibodies against peptide 2 were not successful in their specificity. Signal from the antibody against peptide 2 was observed in cells that were not transfected (left three panels of **figure 6B)**. Antibodies against peptide 1 were modestly more specific (**figure 6A**).

**Supplemental figure 1.** Immunocytochemical results against all three antibodies against peptide 1 (A) and peptide 2 (B).  Parallel experiments were done in cells that were both non-transfected (-) of transfected (+) with the r5-HT _7_ plasmid.  FITC channel visualizes the 5-HT_7_ receptor staining while TRITC is staining for the FLAG epitope in the same cells.  “All channels” is the overlay of the FITC + TRITC channel.  Scale bar bottom right = 50 µm.

**Supplemental figure 2.** Colocalization of the r5-HT_7_ receptor with smooth muscle layer of the rat portal vein.  FITC channel visualizes the 5-HT_7_ receptor staining while TRITC is staining for smooth muscle α-actin “All channels” is the overlay of the FITC + TRITC channel.  Scale bar bottom right = 50 µm . Representative of four (4) different animals. White arrows point to the place of staining/colocalization as the region of interest in this experiment (expression in smooth muscle).
