# Supplementary figures and images for: Development of a 5-HT7 receptor antibody for the rat: the good, the bad, and the ugly
Source: Naunyn Schmiedebergs Arch Pharmacol. 2023 Apr 18;396(10):2599–611. doi: 10.1007/s00210-023-02482-w (PMC10497691; doi:10.1007/s00210-023-02482-w)

A.

## Peptide 1 Antibodies

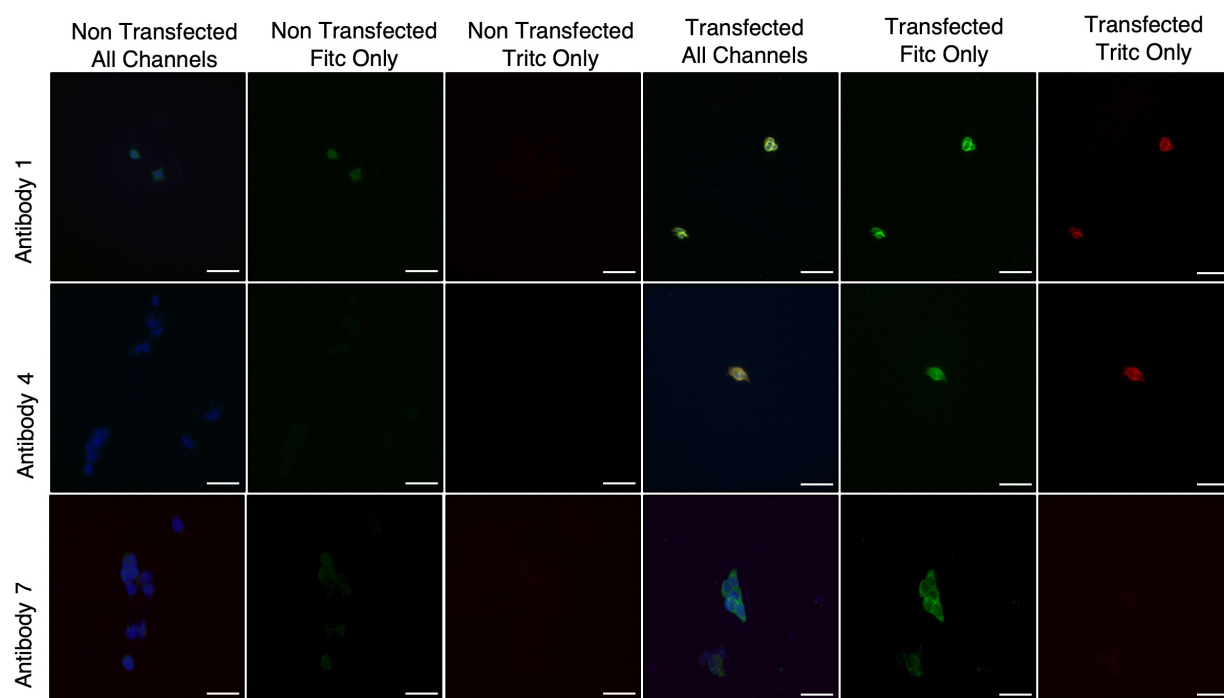

## Peptide 2 Antibodies

B.

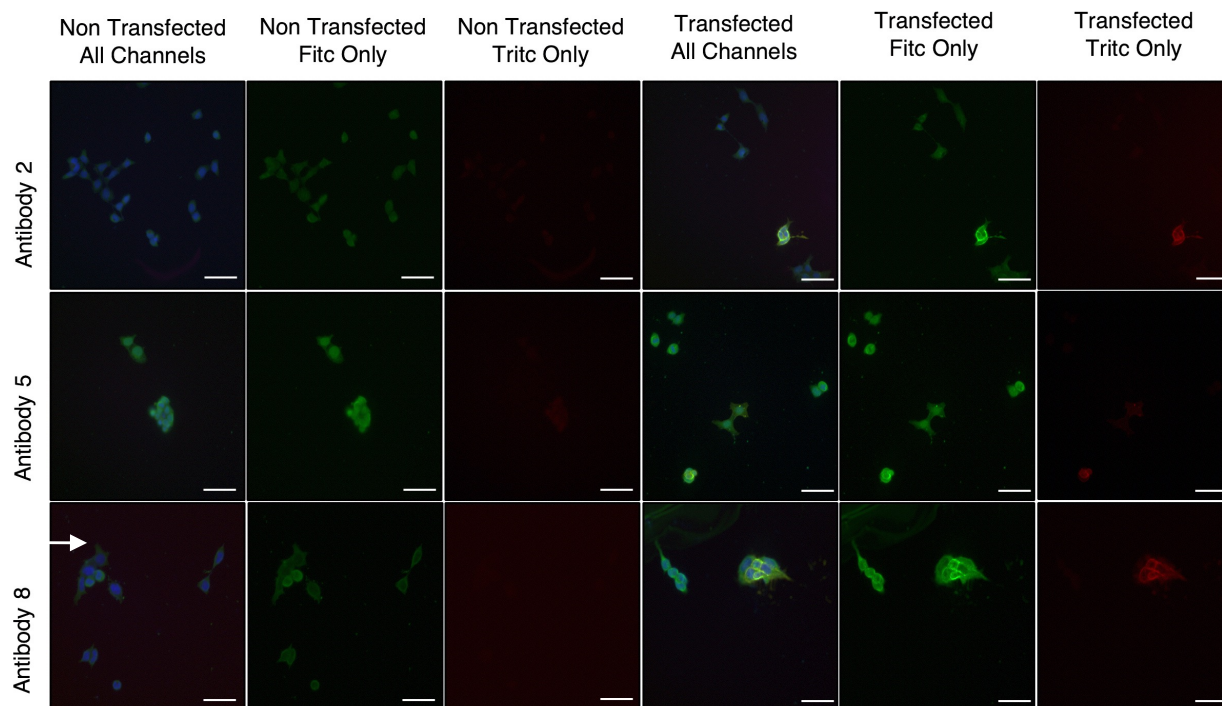

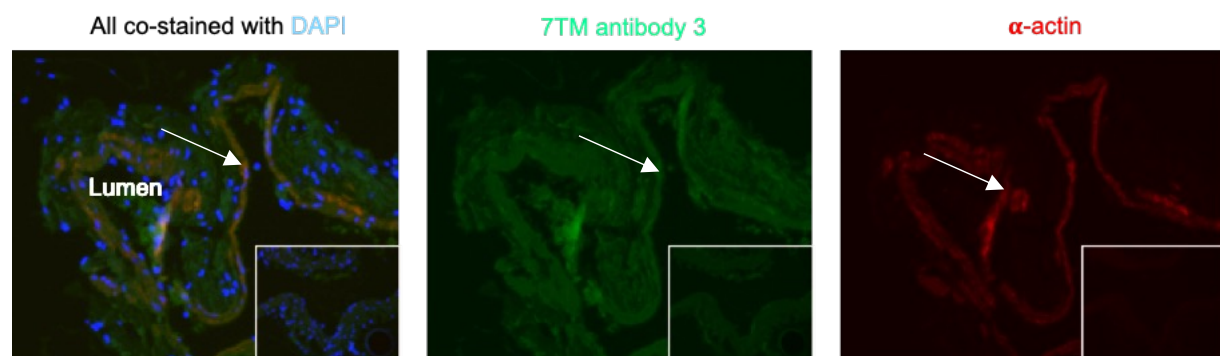

Supplemental Fig 2

Supplement: Supplementary file 2 — Supplementary file2 (PDF 1128 kb) [file 210_2023_2482_MOESM2_ESM.pdf]
